# Pancreatic cancer mortality trends attributable to high fasting blood sugar over the period 1990–2019 and projections up to 2040

**DOI:** 10.3389/fendo.2024.1302436

**Published:** 2024-07-05

**Authors:** Yongguang Wei, Zedong Qin, Xiwen Liao, Xin Zhou, Huasheng Huang, Chenlu Lan, Wei Qin, Guangzhi Zhu, Hao Su, Tao Peng

**Affiliations:** ^1^ Department of Hepatobiliary Surgery, The First Affiliated Hospital of Guangxi Medical University, Nanning, China; ^2^ Guangxi Key Laboratory of Enhanced Recovery After Surgery for Gastrointestinal Cancer, Nanning, China; ^3^ Key Laboratory of High-Incidence-Tumor Prevention & Treatment (Guangxi Medical University), Ministry of Education, Nanning, China; ^4^ Departments of Oncology, Xichang People’s Hospital, Xichang, China

**Keywords:** pancreatic cancer, high fasting plasma glucose, diabetes, global trend, projection

## Abstract

**Background:**

Pancreatic cancer (PC) is a prevalent malignancy within the digestive system, with diabetes recognized as one of its well-established risk factors.

**Methods:**

Data on PC mortality attributed to high fasting blood sugar were retrieved from the Global Burden of Disease (GBD) study 2019 online database. To assess the temporal trends of PC burden attributable to high fasting plasma glucose (HFPG), estimated annual percentage changes (EAPCs) for age-standardized death rates (ASDRs) between 1990 and 2019 were determined using a generalized linear model. Furthermore, a Bayesian age-period-cohort (BAPC) model using the integrated nested Laplacian approximation algorithm was employed to project the disease burden over the next 20 years.

**Results:**

Globally, the crude death number of PC attributable to HFPG almost tripled (from 13,065.7 in 1990 to 48,358.5 in 2019) from 1990 to 2019, and the ASDR increased from 0.36/100,000 to 0.61/100,000 with an EAPC of 2.04 (95% CI 1.91–2.16). The population aged ≥70 years accounted for nearly 60% of total deaths in 2019 and experienced a more significant increase, with the death number increasing approximately fourfold and the ASDR increasing annually by 2.65%. In regions with different sociodemographic indexes (SDIs), the highest disease burden was observed in the high-SDI region, whereas more pronounced increasing trends in ASDR were observed in the low to middle-SDI, low-SDI, and middle-SDI regions. Additionally, a significantly negative association was found between EAPCs and ASDRs of PC attributable to HFPG from 1990 to 2019. Moreover, the BAPC model predicts that ASDR and age-standardized disability-adjusted life-years (DALYs) rate for PC attributed to HFPG was projected to increase obviously for men and women from 2019 to 2040.

**Conclusions:**

The burden of PC attributed to HFPG has increased globally over the past three decades, with the elderly population and high-SDI regions carrying a relatively greater disease burden, but more adverse trends observed in low-SDI areas. Furthermore, the burden is projected to continue increasing over the next 20 years. Hence, more tailored prevention methodologies should be established to mitigate this increasing trend.

## 1 Introduction

Pancreatic cancer (PC), with a 5-year overall survival rate of only 4%–12%, presents one of the highest mortalities among all solid tumors (1, 2), particularly in developed nations and regions. Risk factors for PC, including inherited genetics, smoking, obesity, diabetes, and excessive drinking (1, 3), have been identified. Despite significant advancements in addressing PC over the past decades (4), its incidence continues to rise globally (3). This unfavorable trend is attributed to population expansion, aging populations, and changes in prevalent cancer-related risk factors, many of which are associated with societal and economic development. The growing prevalence of diabetes exerts a significant burden on society in terms of mortality, morbidity, and socioeconomic costs (5). Remarkably, the increasing prevalence of diabetes and obesity also contributes to the increase of PC incidence (6–8). Given the compelling links between diabetes and cancers (9, 10), the pandemic of diabetes portends an increase in the number of cancer diagnoses, including PC.

Precise prevention of PC can be advanced through targeted prevention approaches informed by a thorough understanding of PC mortality patterns and temporal trends. Adherence to type 2 diabetes mellitus (T2DM) prevention diets has been shown to lower risk of PC in the United States population (11). The Global Burden of Disease (GBD) 2019 study, with a wide range of data sources and scientific computational methodologies, has labored deliberately and systematically to disclose incidence, prevalence, mortality, years of life lost (YLLs), years lived with disability, and disability-adjusted life-years (DALYs) due to 369 diseases and injuries for two sexes, 20 age groups in seven super-regions, 21 regions, and 204 countries and territories, from 1990 to 2019. Currently, some studies have examined the burden of multiple cancers attributable to high fasting plasma glucose (HFPG), the most common indicator of diabetes within the GBD framework (12–14). However, research on the temporal and future trends of PC mortality attributable to HFPG is limited, especially concerning age and geographical dimensions (15, 16).

To explore the global burden of PC attributed to HFPG, we comprehensively analyzed the temporal trends of PC mortality globally and nationally from 1990 to 2019, based on data from GBD 2019. We further projected future disease burdens. These findings not only complement prior research but also offer guidance for designing and promoting targeted prevention strategies for PC attributed to HFPG (17).

## 2 Materials and methods

### 2.1 Data source

The data on annual crude deaths, DALYs, and the corresponding age-standardized rates of PC with “HFPG” from 1990 to 2019, categorized by sex, region, and country, were extracted from the Global Health Data Exchange query tool (http://ghdx.healthdata.org/gbd-results-tool) (18). Diabetes is commonly defined as a serum fasting plasma glucose (FPG) level exceeding 7 mmol/L (19, 20). In GBD 2019, the mean FPG, measured in mmol/L, was identified as the continuous exposure for HFPG and estimated from diabetes prevalence using an ensemble distribution established by GBD 2019. HFPG, as an individual risk factor, was defined as any FPG level beyond the theoretical minimum-risk exposure level (4.8 mmol/L–5.4 mmol/L) by the Institute for Health Metrics and Evaluation. Detailed methodologies have been described in previous publications (6, 12). The sociodemographic index (SDI), reflecting geographic closeness and epidemiological commonality, is a comprehensive indicator of socioeconomic development, ranging from 0 (worst) to 1 (best). SDI data for different countries were also retrieved from GBD 2019 (https://ghdx.healthdata.org/record/ihme-data/gbd-2019-socio-demographic-index-sdi-1950-2019). According to SDI, 204 countries and territories were divided into five areas and 21 regions. To predict the mortality of PC attributable to HFPG globally, the population estimates for 2017–2100 were also obtained from GBD 2019 (https://ghdx.healthdata.org/record/ihme-data/global-population-forecasts-2017-2100).

### 2.2 Estimated annual percentage change

To further quantify the burden trend for PC attributable to HFPG globally and in subgroups, including sex, age, and SDI during 1990–2019, the estimated annual percentage change (EAPC), reflecting the annual change across a specific time period, was calculated (21, 22). We utilized a generalized linear regression model lnR = α + βT + ϵ, where R represents the amount or rate and T represents the calendar year. EAPC was determined as 100 − (exp[β]−1), with a 95% confidence interval (CI). An upward trend in age-standardized death rate (ASDR) occurs when both the EAPC measures and their corresponding lower limits of 95% CIs were > 0. On the contrary, if both EAPC values and their upper limits of 95% CIs were < 0, a downward trend would be indicated. Otherwise, ASDR was considered stable.

### 2.3 Mortality estimates by the Bayesian age-period-cohort model

The prospective trend of a disease may help to establish appropriate health policies and promote the reasonable allocation of medical resources. Bayesian age-period-cohort (BAPC) models, using integrated nested Laplace approximations and assuming an inverse-gamma prior distribution of extant data, offer a practical approach for the prediction of cancer mortality rates (23–25). Using R packages “BAPC” and “INLA (integrated nested Laplace approximation)” and GBD data from 1990 to 2019, the BAPC model was employed to assess mortality and DALY loss due to PC attributed to HFPG globally, projecting future disease burdens until 2040.

### 2.4 Data description and statistical analysis

All tests, calculations, and descriptive plotting were conducted using the R program (v4.1.1). The trend of disease burden across different regions and age groups was also assessed. To categorize temporal ASDR trends for each nation, a hierarchical cluster analysis was conducted, classifying countries and territories into four growth types (significant increase; minor increase; remained stable or minor decrease; significant decrease) based on EAPCs of ASDRs. Additionally, we examined the correlation between EAPCs and ASDRs (1990) globally using Pearson correlation analysis. Furthermore, Pearson rank-order correlation analysis was conducted to determine the strength and direction of the connection between SDI and ASDR at the regional and national levels, respectively. Unless otherwise indicated, a significance level of *P* < 0.05 was applied as the threshold cutoff value.

## 3 Results

### 3.1 Temporal trends of crude deaths, DLAYs, and their corresponding ASDRs for PC attributed to HFPG at the global level from 1990 to 2019

**Figure 1:**
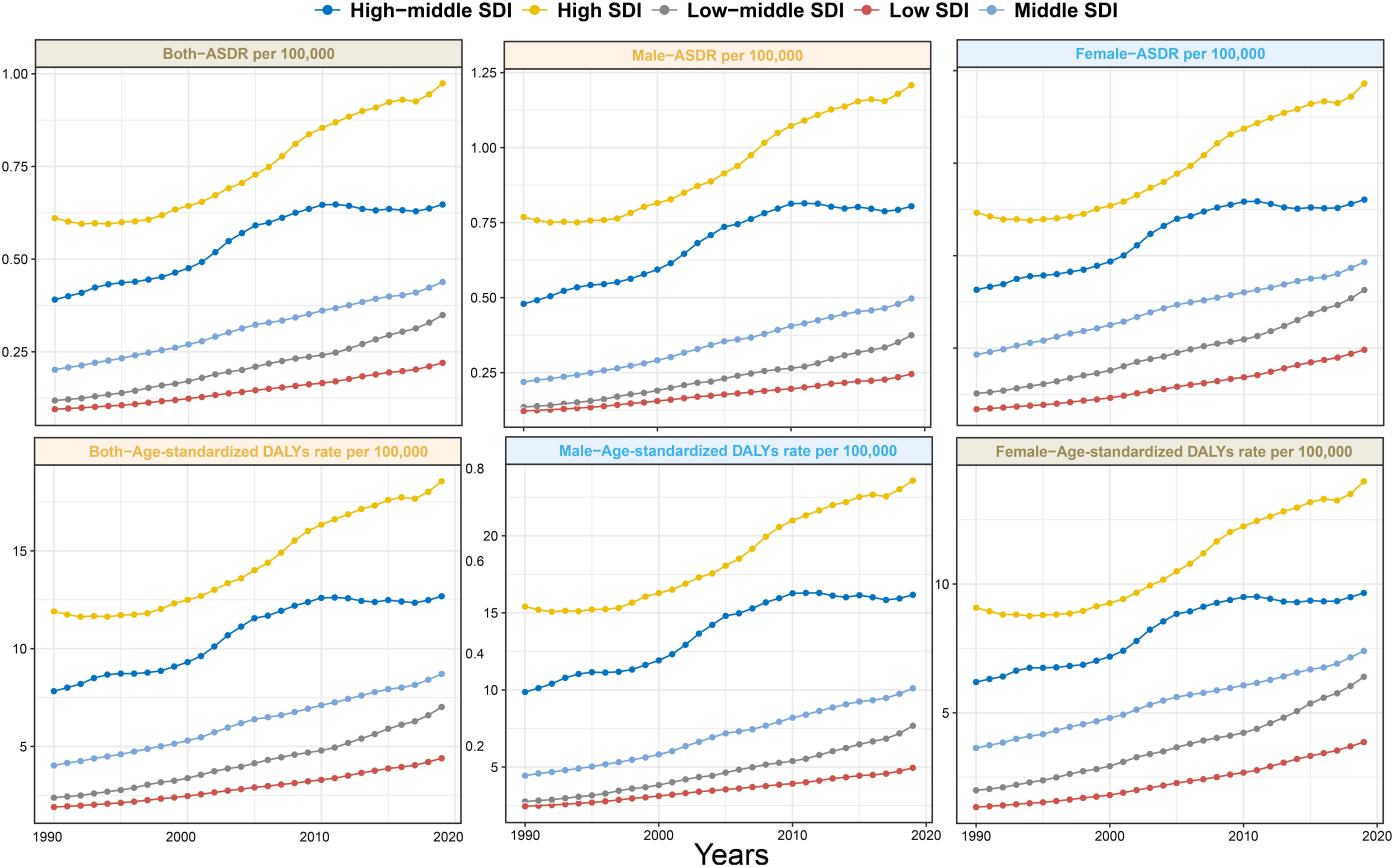
Trends in ASDR and age-standardized DALYs rate of pancreatic cancer attributed to HFPG among men, women, and both sexes in different SDI regions from 1990 to 2019. ASDR, age-standardized death rate; DALYs, disability-adjusted life-years; HFPG, high fasting plasma glucose; SDI, sociodemographic index.

**Figure 2:**
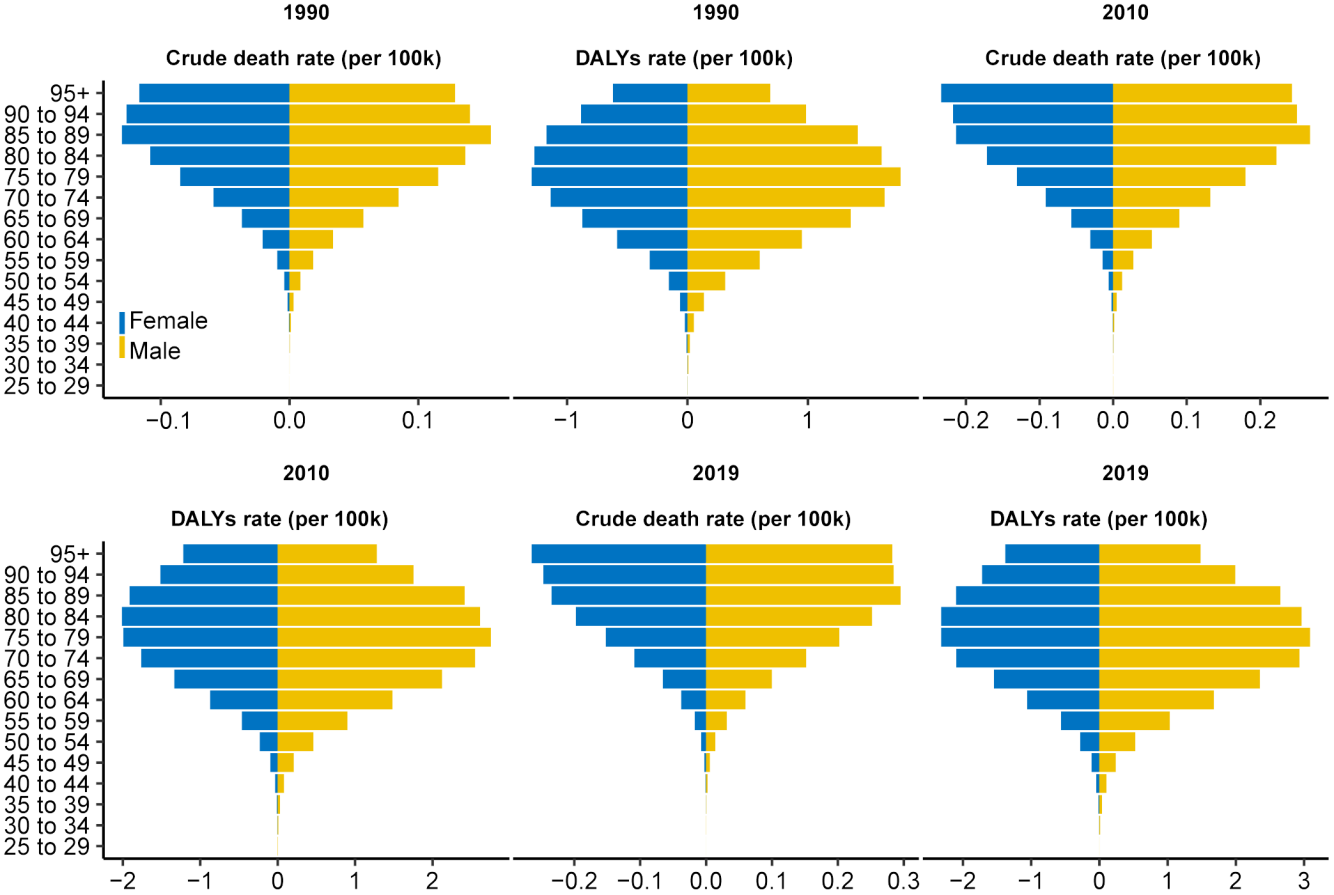
Distribution of ASDR and age-standardized DALY rate for pancreatic cancer attributed to HFPG by age group in 1990, 2010, and 2019. The horizontal axis represents the ASDR and age-standardized DALYs rate (per 100,000 persons), whereas the vertical axis represents different age groups. The blue stripe on the left represents women, whereas the yellow stripe on the right represents men. ASDR, age-standardized death rate; DALYs, disability-adjusted life-years; HFPG, high fasting plasma glucose.

The crude death number of PC attributed to HFPG increased nearly threefold (from 13,065.7 in 1990 to 48,358.5 in 2019) from1990 to 2019 globally, accompanied by an increase in the ASDR from 0.36/100,000 to 0.61/100,000, with a global EAPC value of 2.04 (95% CI 1.91–2.16. That is to say, approximately 0.61% of all PC-related deaths in 2019 were attributed to HFPG (**Table 1**, **Figure 1**). Similarly, among the five SDI regions, an obvious increasing trend in crude deaths and ASDRs was observed across all age groups for men, women, and both sexes (**Table 1**, **Figure 1**). The increasing trends in ASDRs and DALYs for both sexes, women, and men were nearly consistent (**Figure 1**). Each gender contributed roughly half of all deaths. Although crude death number and ASDRs increased across all three age subgroups (**Table 1**), the subgroup aged ≥70 years, constituting nearly 60% of total deaths in 2019, experienced a more obvious increase in contrast to other age groups. Specifically, crude deaths in this subgroup increased approximately fourfold, with an annual ASDR increase of 2.65% between 1990 and 2019. In terms of SDI regions, the high SDI region had the highest ASDR in 2019 (0.97 [95% CI 0.23–2.07]), with ASDRs decreasing as SDI decreased. Despite that the high SDI region had a greater disease burden, more pronounced increasing trends in ASDRs were observed in the low to middle-SDI (EAPC: 3.75 [95% CI 3.67–3.82]), low-SDI (EAPC: 2.98 [95% CI 2.93–3.02]), and middle-SDI (EAPC: 2.73 [95% CI 2.63–2.83]) regions (**Table 1**, **Figure 1**). The distribution of ASDR and DALY rates across different age subgroups was largely consistent for men and women in 1990, 2010, and 2019 (**Figure 2**). In addition, the distribution of crude DALY rates peaked in the 75–79 age group and experienced an obvious rise across all SDI regions from 1990 to 2019 (**Figure 2**; **Supplementary Figure 1**). Conversely, crude death rate distributions in different age groups were unimodal for men in 1990, 2010, and 2019, and for women in 1990, with crude death rates peaking in the 85–95 age group. In addition, the crude death rate for women in 2010 and 2019 showed a skewed distribution, with a peak in the 95+ age group (**Figure 2**).

**Table 1:** The changing trends of number of deaths and age-standardized mortality rate of pancreatic cancer among patients with type 2 diabetes from 1990 to 2019.

	1990		2019		1990–2019
	Death cases no. (95% UI)	ASDR per 100,000 no. (95% UI)	Death cases no. (95% UI)	ASDR per 100,000 no. (95% UI)	EAPC no. (95% CI)
Overall	13,065.7 (2,985.2–28,677.0)	0.36 (0.08–0.799)	48,358.5 (11,539.6–103,691.0)	0.61 (0.15–1.30)	2.04 (1.91–2.16)
Sex					
Female	6,359.1 (14,835.9–1,048.8)	0.24 (0.56–0.04)	22,417.4 (52,358.4–3,741.3)	0.58 (1.36–0.10)	1.90 (1.77 to 2.02)
Male	6,706.7 (15,795.0–1,213.2)	0.25 (0.59–0.05)	25,941.1 (59,099.0–4,858.8)	0.67 (1.52–0.13)	2.15 (2.01 to 2.28)
Age (years)					
15–49	319.0 (66.4–745.9)	0.01 (0.002–0.03)	1,058.8 (222.9–2,496.2)	0.03 (0.01–0.06)	2.10 (2.92–3.05)
50–69	5,123.2 (1,139.8–11,521.2)	0.75 (0.17–1.69)	17,048.9 (3,908.0–3,7312.3)	1.24 (0.28–2.71)	1.86 (1.77–1.95)
≥70	7,623.5 (1,735.4–16,611.6)	3.78 (0.86–8.24)	30,250.8 (7,339.8–64,503.3)	6.52 (1.58–13.91)	2.19 (2.06–2.31)
SDI					
High SDI	6,554.7 (1,505.8–14,352.3)	0.61 (0.14–1.34)	19,644.7 (4,755.0–41,729.5)	0.97 (0.23–2.07)	2.00 (1.84–2.17)
High-middle SDI	3,945.0 (892.0–8,669.3)	0.39 (0.09–0.86)	13,214.5 (3,078.4–28,804.6)	0.65 (0.15–1.41)	1.97 (1.73–2.22)
Middle SDI	1,774.6 (391.9–3,888.5)	0.20 (0.05–0.44)	10,140.2 (2,341.5–21,943.4)	0.44 (0.10–0.95)	2.73 (2.63–2.83)
Low-middle SDI	597.6 (127.3–1,407.2)	0.12 (0.03–0.28)	4,361.4 (1,031.3–9,457.5)	0.35 (0.08–0.76)	3.75 (3.67–3.82)
Low SDI	186.6 (38.3–455.6)	0.10 (0.02–0.23)	971.1 (221.8–2,173.3)	0.22 (0.05–0.49)	2.98 (2.93–3.02)

ASDR, age-standardized death rate; SDI, sociodemographic index; EAPC, estimated average percentage change; UI, uncertainty interval; CI, confidence interval.

### 3.2 Global disease burden assessment of PC attributed to HFPG for different countries in 2019

**Figure 3:**
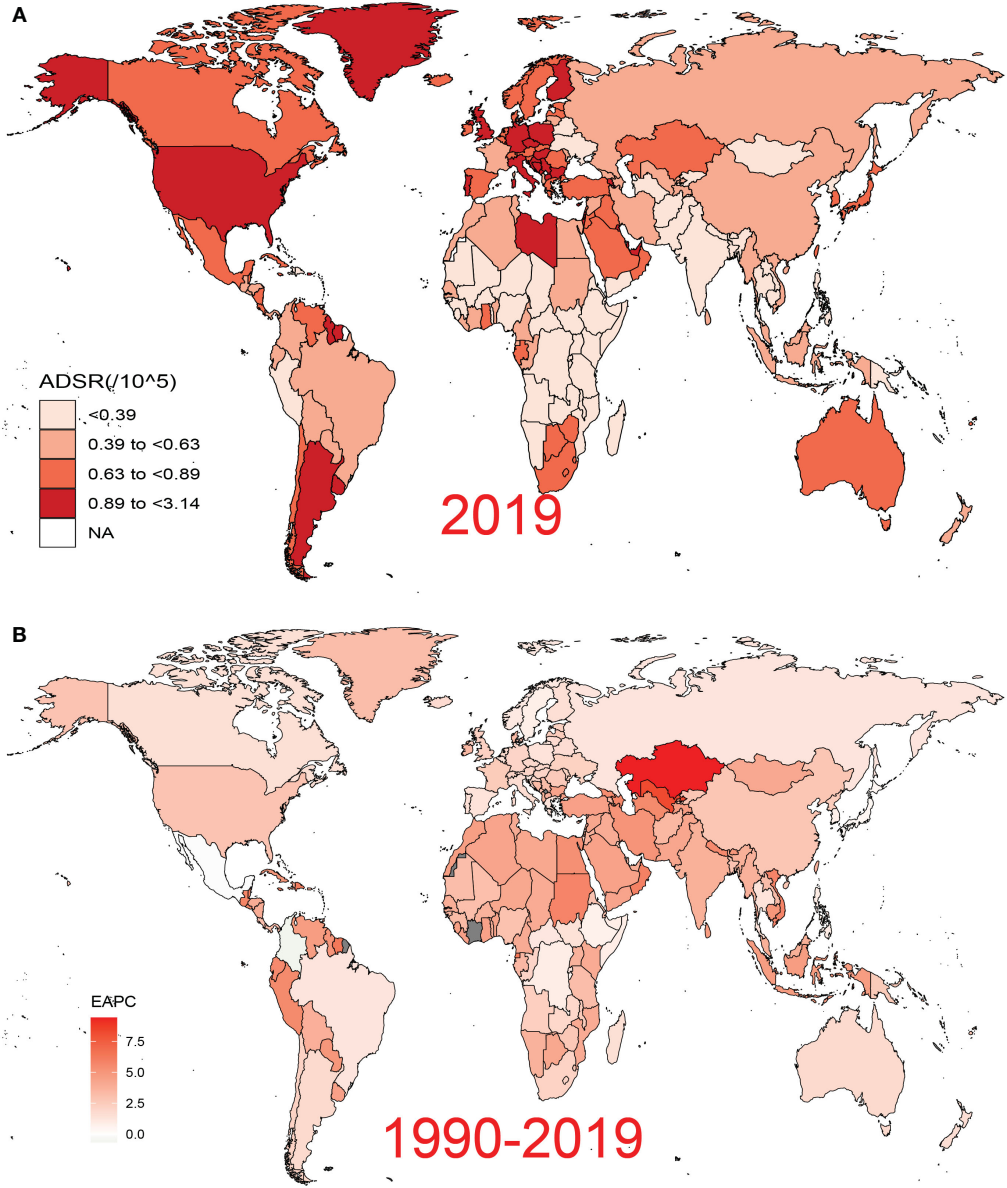
Global disease burden of pancreatic cancer attributed to HFPG for both sexes across 195 countries and territories. **(A)** ASDR of pancreatic cancer attributed to HFPG in 2019. **(B)** EAPC of ASDR for pancreatic cancer attributed to HFPG from 1990 to 2019. HFPG, high fasting plasma glucose; ASDR, age-standardized death rate; EAPC, estimated annual percentage change.

*In 2019*, both crude death and DALY rats exhibited an increasing trend not only from young to old individuals but also from high to low SDI regions (**Supplementary Figure 2**). In addition, the ratio of male to female crude death rates across different SDI regions showed a generally consistent distribution across different age groups globally, with the main peak occurring in the 30–40 age group (**Supplementary Figure 3**). Moreover, from the 35–39 age group onward, the ratio showed a decreasing trend but remained above 1 with increasing age. The world map showed highly heterogeneous ASDRs and corresponding EAPCs for PC attributed to HFPG across different countries and territories in 2019 (**Figure 3**). Particularly severe ASDRs were observed in a few countries bordering the Mediterranean, as well as in most countries in North America, Europe, and South America (**Figure 3A**). Based on EAPC values, almost all countries experienced varying degrees of increase in disease burden from 1990 to 2019, with more pronounced increases primarily detected in developed countries, reaching a peak in Kazakhstan and Uzbekistan. In cluster analysis, 204 countries and territories were categorized into four subgroups with different increasing rates: significant increase, increase, remained stable or minor decrease, and significant decrease (**Supplementary Figure 4**).

### 3.3 The influential factor for EAPCs of ASDR for PC attributable to HFPG

**Figure 4:**
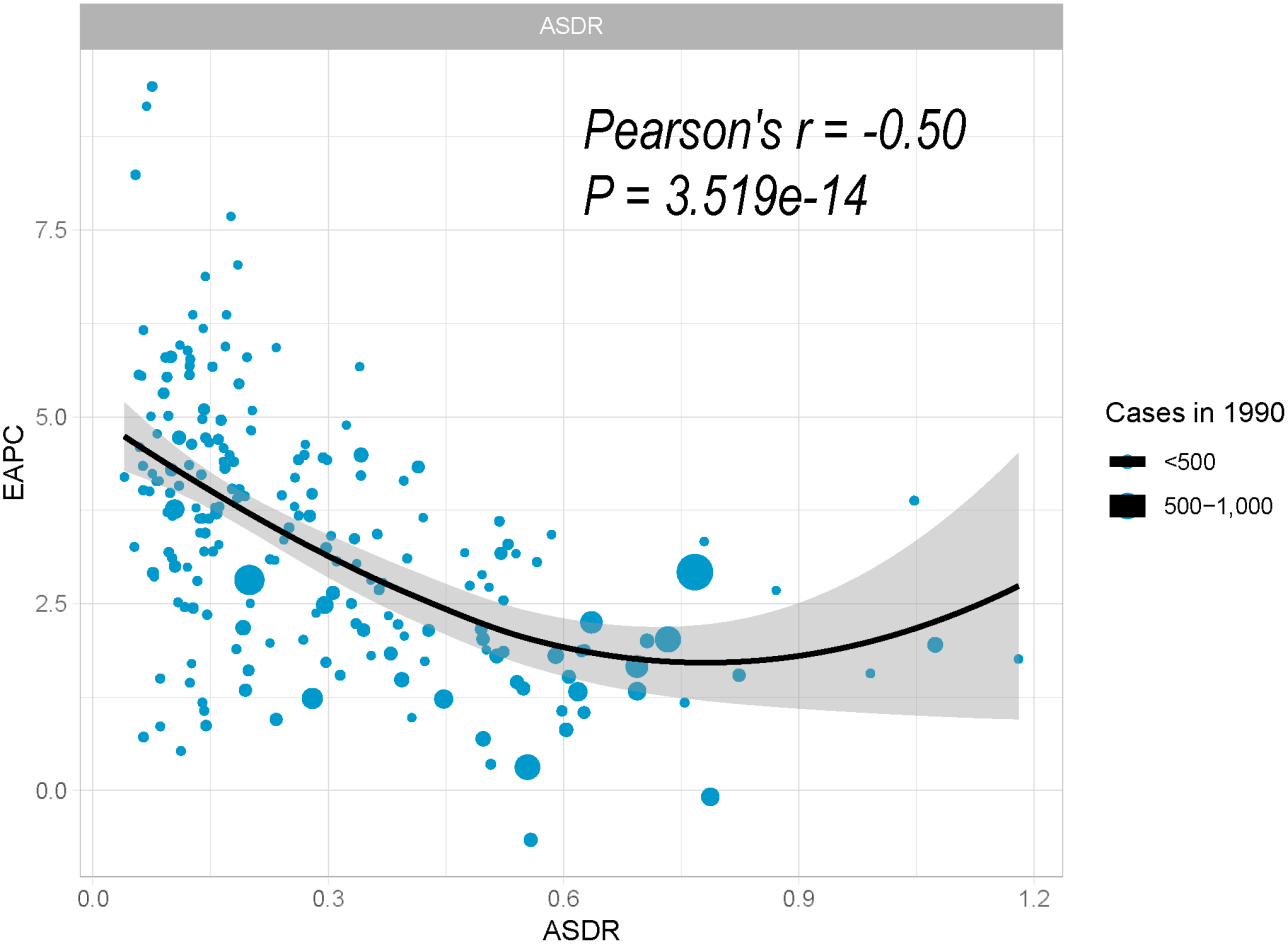
Correlation between EAPC and ASDR of pancreatic cancer attributed to HFPG in 1990. The size of the circle is proportional to the number of pancreatic cancer cases. EAPC, estimated annual percentage change; ASDR, age-standardized death rate; HFPG, high fasting plasma glucose.

The baseline disease pool of PC attributable to HFPG is represented by the ASDRs in 1990. In our study, significantly negative associations were found between EAPCs and ASDRs (**Figure 4**; Pearson’s r = −0.50; *P* < 0.001).

### 3.4 The trend in ASDR for PC attributed to HFPG across SDI by region or nation from 1990 to 2019

**Figure 5:**
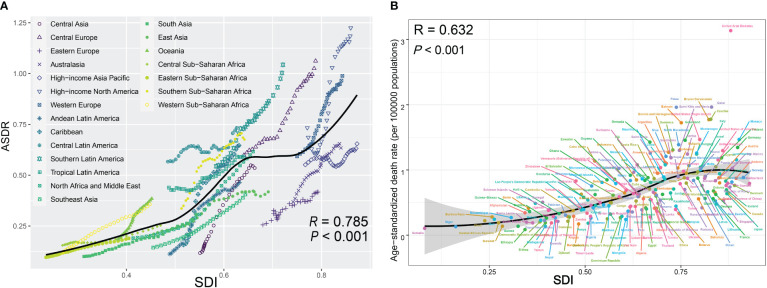
**(A)** Trend in ASDRs of pancreatic cancer attributed to high fasting plasma glucose (HFPG) among/across 21 regions based on SDI in 2019. For each region, points from left to right depict estimates from each year from 1990 to 2017, with expected values shown as the black line. **(B)** ASDRs of pancreatic cancer attributed to HFPG across 195 countries and territories by SDI in both sexes, 2019. Expected values are shown as the black line. Each point shows observed age-standardized DALY rate for a certain country in 2019. ASDRs, age-standardized death rates; HFPG, high fasting plasma glucose; SDI, sociodemographic index.

Among the 21 regions of different SDI, most regions exhibited ascending trends of ASDRs along with increases in SDI from 1990 to 2019 (**Figure 5A**; *R* = 0.785; *P* < 0.001). In this context, most high-income regions exceeded the expected level in all years, whereas many low-income regions, despite experiencing an upward trend, remained below the expected level (**Figure 5A**). **Figure 5B** illustrates the connection between ASDRs and SDI across different countries and territories in 2019. There was also a general upward tendency of ASDRs along with increases in SDI at the national level, which was similar to regional pattern. However, this upward trend leveled off after SDI exceeded 0.7 (**Figure 5B**; *R* = 0.632; *P* < 0.001).

### 3.5 Global disease burden prediction for PC attributable to HFPG

**Figure 6:**
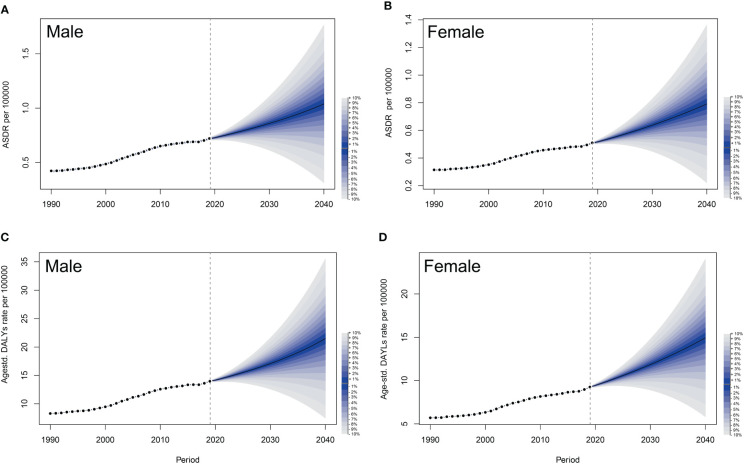
Observed and predicted trends of ASDRs and age-standardized DALYs of pancreatic cancer attributed to HFPG by sex globally from 1990 to 2040 using the BAPC model. **(A)** ASDRs of male. **(B)** ASDRs of female. **(C)** Age-standardized DALYs of male. **(D)** Age-standardized DALYs of female. The shadow in the figure represents uncertainty intervals, suggesting that mortality could fluctuate dramatically as the corresponding rates rise or fall by 1% per year, with each shade corresponding to a change of 1%. ASDRs, age-standardized death rates; DALYs, disability-adjusted life-years; HFPG, high fasting plasma glucose; BAPC, Bayesian age-period-cohort; UIs, uncertainty intervals.

Based on the comprehensive GBD data from 1990 to 2019, we utilized the BAPC model (26) to project that both ASDR and age-standardized DALY rates for PC attributed to HFPG would significantly increase for both sexes over the period 2019–2040 (**Figures 6A–D**). The shaded areas in the figure indicate that the mortality could fluctuate dramatically as the corresponding rates rise or fall by 1% per year.

## 4 Discussion

T2DM is a significant contributing factor to PC (27). Insulin resistance, induced by either a paraneoplastic syndrome or pancreatic β-cell dysfunction, serves as a pivotal mechanism in the development of diabetes mellitus (DM) (28). In the pathogenesis of PC induced by DM, exosomes and miR-19a emerge as crucial mediators and effector molecules (29). Furthermore, there are several potential driving mechanisms in the proliferation and invasion of PC cells, including activation of the high glucose-activated p38 MAPK pathway, enhanced expression of *GDNF* and *RET*, induction of *EGF* expression, and *EGFR* transactivation (28, 30, 31). The high-glucose microenvironment in PC can further promote cancer development by affecting the *SREBP1*–autophagy axis (32). In addition, type 3c diabetes, also known as pancreatogenic diabetes, which arises when the pancreas suffers chronic injury, may increase the risk of pancreatic ductal adenocarcinoma (33). These findings shed light on the pathogenesis of PC associated with hyperglycemia or DM (34).

Previous studies have shown that age-standardized PC deaths were primarily attributable to smoking (21.1%), HFPG (8.9%), and high body mass index (BMI) (6.2%) worldwide in 2017 (6). The proportion of PC deaths attributed to tobacco consumption declined, whereas those attributed to HFPG and high BMI increased during 1990–2019 (7). Notably, the 65–69 age group had the greatest proportion of incident cases and deaths from PC, whereas the disease burden peaked in the 75–79 age subgroup in women (6). The increasing burden of diabetes brings a cumulative risk of human cancers (10, 27).

Based on global investigation and statistics, the burden of T2DM increased by 1.56% per year (1.64% in men and 1.51% in women) worldwide from 2000 to 2019. The most significant incidence increases took place in the Eastern Mediterranean, the United States of America, and Southeast Asia, whereas the smallest increases were observed in the Western Pacific, Africa, and Europe. The most pronounced increases in age-standardized T2DM prevalence were observed in high-SDI countries, followed by low-medium and low SDI countries (35). Diabetes was more common in individuals over the age of 60 and had no significant sex differences (5). Alarmingly, global estimates predict that the number of adults with impaired glucose tolerance will rise from 374 million in 2017 to 587 million by 2045 (5). Obesity, which can decrease internal sensitivity to insulin, may induce impaired glucose tolerance and even diabetes (36). Moreover, metabolic changes due to obesity and diabetes may directly or indirectly contribute to cancer progression (37). High BMI is causally linked to various cancers, including PC (38, 39). Cumulative risk effects for developing PC may arise from diabetes accompanied by weight loss (40).

In our study, we demonstrated an increasing trend in the global disease burden for PC mortality attributed to HFPG from 1990 to 2019, and this trend is expected to continue over the next 20 years in both sexes. Furthermore, we found that the death burden was significantly concentrated in the elderly population, which also experienced a more pronounced increase. Additionally, the mortality difference between men and women was not obvious, which was similar to the prevalence characteristics of diabetes (5). Moreover, higher SDI regions had higher ASDRs of PC attributed to HFPG from 1990 to 2019. This could result from factors such as aging populations, inherited conditions, and lifestyles with long-term exposure to risk factors more prevalent in high SDI countries, including obesity or malnutrition, alcohol abuse, and high-calory diets. The Western diet, comprising high red meat, refined grains, and sugar-sweetened beverages, may contribute to enhancing systemic inflammation agents and, consequently, developing PC (28). However, our study observed more pronounced increasing trends in ASDRs in low to middle-SDI, low-SDI, and middle-SDI regions. In fact, in the Asia-Pacific region, particularly in nations experiencing rapid economic development, the burden of PC is also substantially increasing (41). The variation in increasing trends could stem from exposure to environmental risk factors and lifestyle transformations in the context of globalization. Moreover, the lack of effective screening techniques and poorer medical conditions in low-SDI and middle-SDI countries should also be considered (3, 6, 28, 42). Globally, 87.5% of all undiagnosed diabetes cases occur in low- and middle-income countries, with low-income countries accounting for the largest proportion (50.5%), and even in high-income countries, approximately 28.8% of those with diabetes remain undiagnosed (5).

The present study had several limitations. Firstly, our data source was derived from the GBD 2019, an online database providing predictive information on disease burden, rather than actual data from monitoring and surveillance. Secondly, the etiology of PC is complex, and single attribution is often inadequate to explain the development of this malignancy, despite GBD 2019 excluding other risk factors when processing HFPG exposure data. Thirdly, the projection of disease burden for PC attributable to HFPG relied on mathematical algorithms, and our findings require confirmation through actual epidemiological investigation and data generalization.

Fortunately, the major risk factors related to PC are potentially modifiable, offering an opportunity to prevent this deadly cancer. Positive lifestyle interventions have been shown to delay the onset of diabetes by 4 years, cause all-cause death by 4.82 years, and increase average life expectancy by 1.44 years in populations with impaired glucose tolerance (36). Additionally, intentional weight loss may help prevent the onset of cancer, and disease control of diabetes could serve as a resultful adjuvant to inhibit tumor progression (37). Changes in disease attribution affect the direction of health promotion efforts. Our study emphasized the importance of addressing HFPG, prompting policymakers to allocate resources more efficiently for early diagnosis and reduction of this modifiable risk factor for PC. Strategies to prevent and ameliorate the unhealthy status of HFPG through dietary adjustments, appropriate daily exercise, and medication use as appropriate could help mitigate the risk of PC.

## 5 Conclusions

In conclusion, the burden of PC attributed to HFPG has increased globally over the past three decades, with the elderly population and high-SDI regions bearing a relatively greater disease burden. However, more unfavorable trends were observed in low-SDI areas. Moreover, the burden is projected to continue increasing over the next 20 years. Thus, tailored methodologies should be established for prevention to mitigate this increasing trend.

## Data availability statement

The original contributions presented in the study are included in the article/**Supplementary Material**. Further inquiries can be directed to the corresponding author.

## Author contributions

YW: Data curation, Investigation, Methodology, Resources, Software, Validation, Visualization, Writing – original draft, Writing – review & editing. ZQ: Conceptualization, Data curation, Methodology, Software, Validation, Visualization, Writing – original draft, Writing – review & editing. XL: Data curation, Formal analysis, Software, Validation, Visualization, Writing – original draft, Writing – review & editing. XZ: Investigation, Project administration, Resources, Writing – original draft, Writing – review & editing. HH: Methodology, Software, Validation, Visualization, Writing – original draft. CL: Investigation, Software, Validation, Writing – review & editing. WQ: Formal analysis, Visualization, Writing – review & editing. GZ: Conceptualization, Data curation, Project administration, Writing – review & editing. HS: Data curation, Project administration, Writing – review & editing. TP: Conceptualization, Project administration, Writing – review & editing.
