# Supplementary material for: Pancreatic cancer mortality trends attributable to high fasting blood sugar over the period 1990–2019 and projections up to 2040
Source: Front Endocrinol (Lausanne). 2024 Jul 5;15:1302436. doi: 10.3389/fendo.2024.1302436 (PMC11257875; doi:10.3389/fendo.2024.1302436)
Supplement: Supplementary file 1 [file DataSheet_1.pdf]

Supplementary File-1

Figures and Figure Legends

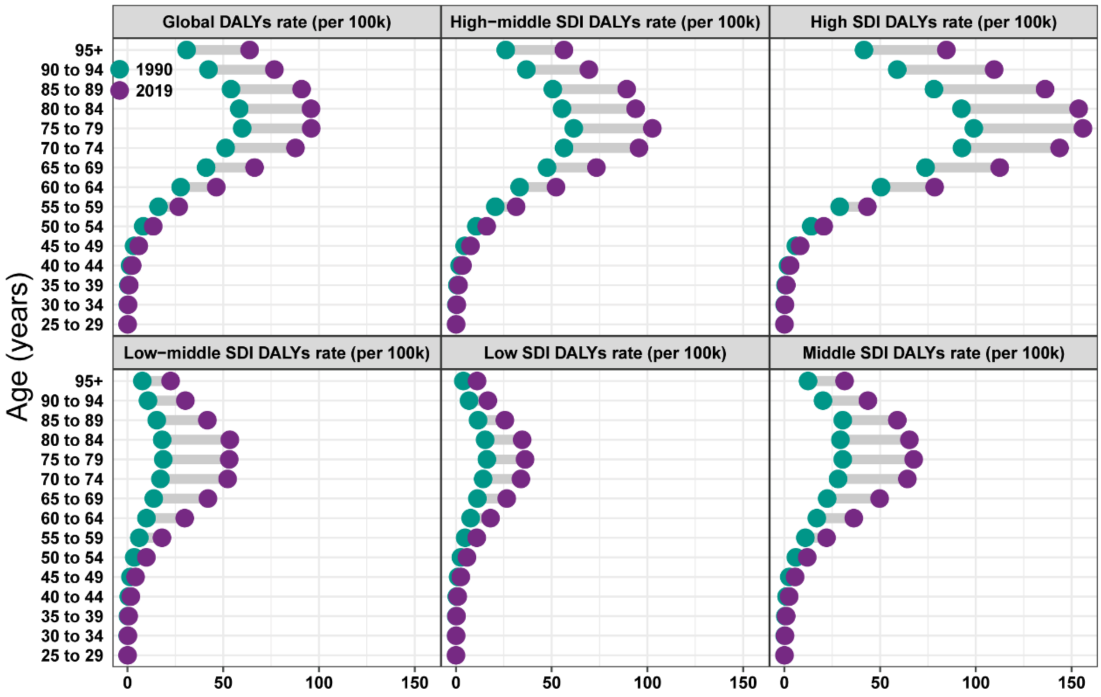

Supplementary Figure S1. The dumbbell chart shows the change in the crude DALYs rate of various age groups for pancreatic cancer attributed to HFPG in different regions from 1999 to 2019. DALYs, disability-adjusted life-years; HFPG, high fasting plasma glucose.

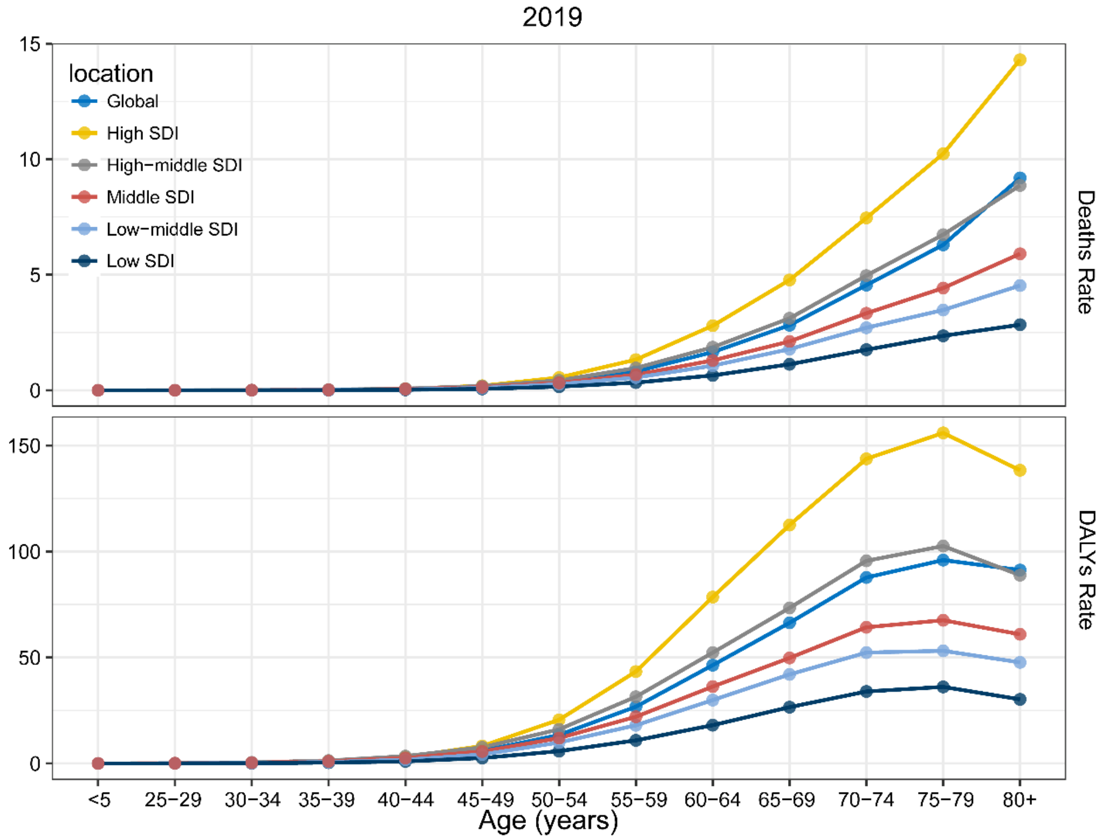

Supplementary Figure S2. Temporal trend of crude deaths rate and DALYs rate of pancreatic cancer attributed to HFPG from young to old people. in different SDI regions. Different colored curves represent the results of different regions. DALYs, disability-adjusted life-years; HFPG, high fasting plasma glucose; SDI, socio-demographic index.

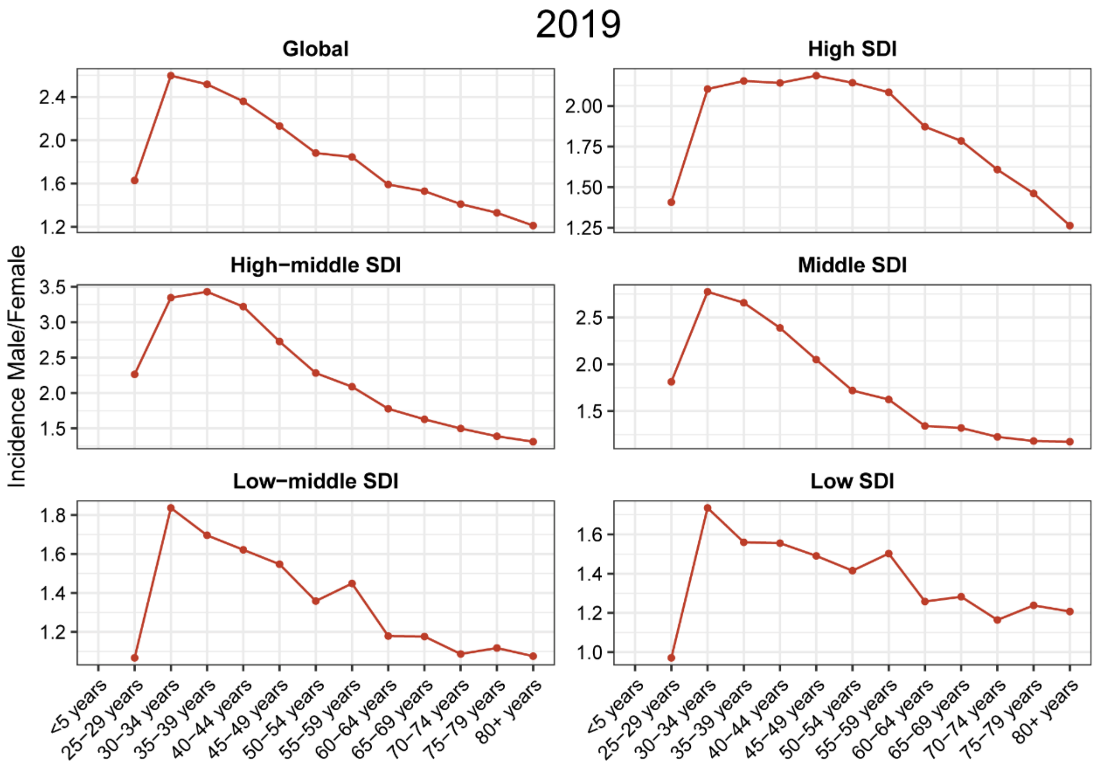

Supplementary Figure S3. The ratio of male to female crude deaths rate for pancreatic cancer attributed to HFPG among various age groups in different SDI regions. ASDR, age-standardized death rate; HFPG, high fasting plasma glucose; SDI, socio-demographic index.

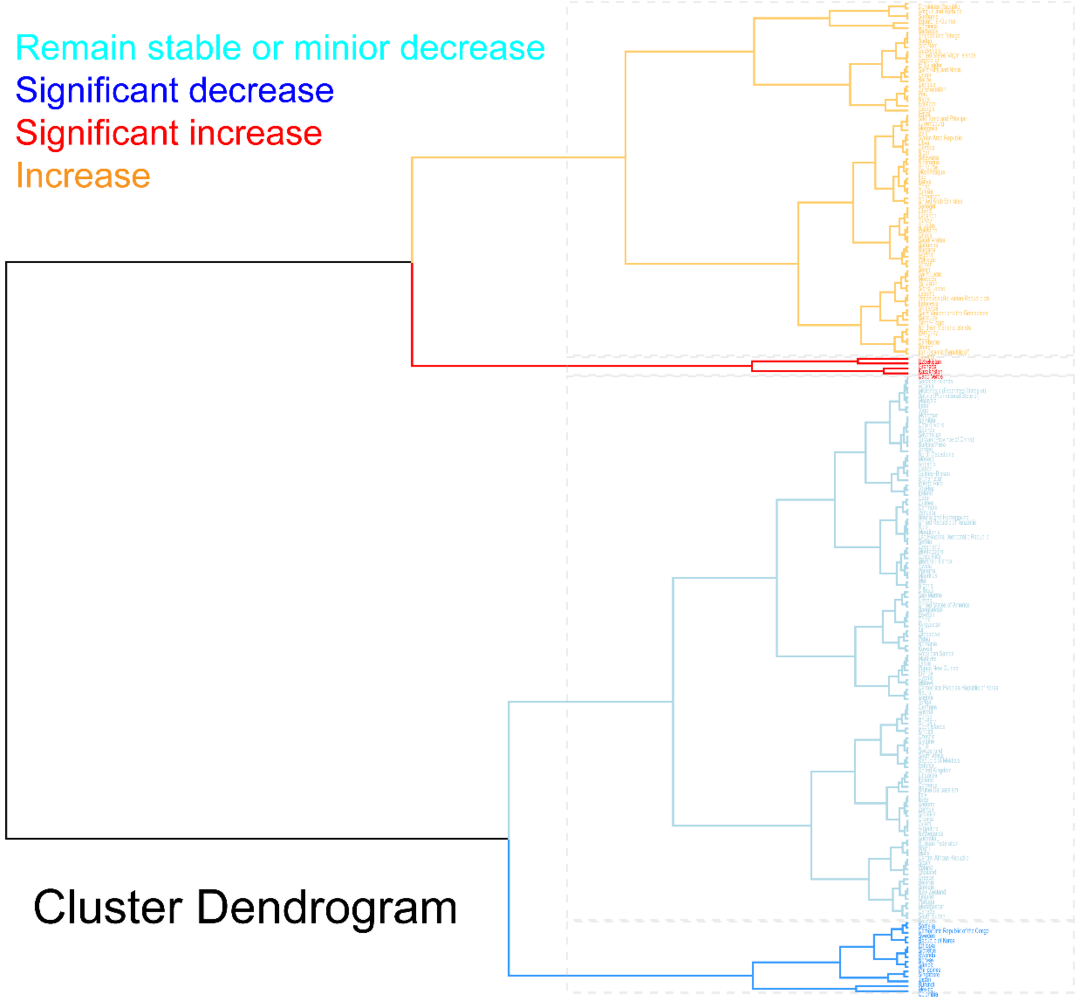

Supplementary Figure S4. Results of cluster analysis based on the EAPC values of the ASDR for pancreatic cancer attributed to HFPG. EAPC, estimated annual percentage change; ASDR, age-standardized death rate; HFPG, high fasting plasma glucose.
